# Enhanced Immune Responses Conferring Cross-Protection by Skin Vaccination With a Tri-Component Influenza Vaccine Using a Microneedle Patch

**DOI:** 10.3389/fimmu.2018.01705

**Published:** 2018-07-30

**Authors:** Wandi Zhu, Song Li, Chao Wang, Guoying Yu, Mark R. Prausnitz, Bao-Zhong Wang

**Affiliations:** ^1^Center for Inflammation, Immunity & Infection, Georgia State University Institute for Biomedical Sciences, Atlanta, GA, United States; ^2^School of Chemical and Biomolecular Engineering, Georgia Institute of Technology, Atlanta, GA, United States; ^3^College of Life Sciences, Henan Normal University, Xinxiang, Henan, China

**Keywords:** microneedle patch, skin vaccination, influenza vaccine, H7N9 influenza virus, immune responses

## Abstract

Skin vaccination using biodegradable microneedle patch (MNP) technology in vaccine delivery is a promising strategy showing significant advantages over conventional flu shots. In this study, we developed an MNP encapsulating a 4M2e-tFliC fusion protein and two types of whole inactivated influenza virus vaccines (H1N1 and H3N2) as a universal vaccine candidate. We demonstrated that mice receiving this tri-component influenza vaccine *via* MNP acquired improved IgG1 antibody responses with more balanced IgG1/IgG2a antibody responses and enhanced cellular immune responses, including increased populations of IL-4 and IFN-γ producing cells and higher frequencies of antigen-specific plasma cells compared with intramuscular injection. In addition, stronger germinal center reactions, increased numbers of Langerin-positive migratory dendritic cells, and increased cytokine secretion were observed in the skin-draining lymph nodes after immunization with the tri-component influenza MNP vaccine. The MNP-immunized group also possessed enhanced protection against a heterologous reassortant A/Shanghai/2013 H7N9 (rSH) influenza virus infection. Furthermore, the sera collected from 4M2e-tFliC MNP-immunized mice were demonstrated to have antiviral efficacy against reassortant A/Vietnam/1203/2004 H5N1 (rVet) and A/Shanghai/2013 H7N9 (rSH) virus challenges. The immunological advantages of skin vaccination with this tri-component MNP vaccine could offer a promising approach to develop an easily applicable and broadly protective universal influenza vaccine.

## Introduction

Influenza virus infection results in high morbidity and mortality in most flu seasons (1–3). Although influenza affects all humans, some groups—including the elderly, infants, children under 5 years old, pregnant women, and people with chronic diseases—are much more vulnerable to influenza virus infection and have increased mortality rates (4–6). Vaccination has been proven to be an effective way to prevent influenza virus infection. However, the protective efficacy of the seasonal flu vaccines is greatly compromised by the high antigenic variation of flu. For instance, the influenza pandemic outbreak in 2009–2010 occurred because of the antigenic drift of an influenza strain rendering the seasonal vaccine ineffective (7). The 2017–2018 flu season demonstrated a widespread outbreak of flu in most of the US. The overall vaccine effectiveness in the 2017–2018 season was 36% overall and only 25% against the H3N2 virus (8). In addition, the highly pathogenic avian influenzas H5N1 and H7N9 have infected humans with high fatality rates in recent years, indicating the possibility of new influenza pandemics in the future.

The first human infection by H7N9 was reported in 2013 and has since caused five seasonal outbreaks and a total of 1,223 infections in humans with a ~40% mortality rate (9, 10). Furthermore, it is reported that the latest H7N9 isolates have diverged into two lineages, the Yangtze River Delta and Pearl River Delta, in which only the latter lineage seems to be sensitive to the existing H7N9 vaccines. This is an example where genetic drift has resulted in a mismatch between the strains in the wild and the potential vaccine strain and reduced our overall preparedness for a flu pandemic. New candidate vaccine strains will need to be included in the H7N9 vaccine formulations to ensure protection.

As the outcome of molecular evolution, H7N9 virus continuously adapts and grows in mammalian species (11–14). Some other mutations have occurred when facing therapeutic pressure (15, 16). The newly emerging H7N9 virus displays increased resistance to neuraminidase inhibitors and poor clinical treatment outcomes. Increased human infections by H7N9 influenza viruses and its rapid divergence have raised concerns and increased interest in the development of broadly protective and rapidly dispersible influenza vaccines.

The promise and effectiveness of skin vaccination is being recognized by researchers, enabled by the novel technology of dissolving microneedle patches (MNPs) (17–20). Vaccines can be delivered by MNP into the epidermis and dermis of the skin, which is a promising vaccination site harboring abundant lymphatic vessels and many different types of immune cells. These skin-migrated and -resident leukocytes are important inducers of the innate immune response and regulators of adaptive immunity (21, 22).

In a recent study, we designed a 4M2e-tFliC construct in which we replaced the hyperimmunogenic region of FliC with four M2e sequences from different influenza subtypes. We found that an M2e-tFliC encapsulated MNP-boosting skin immunization could rapidly broaden the immunity generated by a conventional influenza vaccine prime to confer cross-protection against heterologous virus challenge (23).

M2 is a conserved surface antigen and a promising target for the development of universal influenza vaccines. Results of ours and others have demonstrated that vaccines using recombinant tandem M2e sequences containing the human consensus sequence or diverse sequences from multiple species provided broader protection against influenza virus challenges (16–22). M2e vaccines were found to synergize the efficacy of other influenza vaccines. Therefore, encapsulating M2e vaccines and conventional influenza vaccines into a single MNP is a promising and convenient vaccine strategy to provide broad protection against influenza virus infection.

In this study, we investigated whether skin vaccination with MNPs encapsulating 4M2e-tFliC fusion protein and a divalent inactivated vaccine (DIV) (A/Aichi/2/68, H3N2, Aichi, and A/PR/8/34, H1N1, PR8) can induce increased innate and adaptive immune responses capable of cross-protection against a reassortant H7N9 virus infection.

## Materials and Methods

### Ethics Statement

All animal experiments were performed in accordance with the protocol (protocol number A16029) approved by Georgia State University’s Institutional Animal Care and Use Committee (IACUC). This study was in strict compliance with the Animal Welfare Act Regulations, the Public Health Service (PHS) Policy on Humane Care and Use of Laboratory Animals, and the Guide for the Care and Use of Laboratory Animals.

### Immunization and Challenge

The 4M2e-tFliC fusion protein was purified and identified as previously described (23, 24). For animal studies, two groups of 6- to 8-week-old female BALB/c mice (Harlan Laboratories, Indianapolis, IN, USA) were intramuscularly or MNP skin immunized with tri-component vaccines including 3.2 µg of the 4M2e-tFliC fusion protein, 1.6 µg of HA equivalent Aichi, and 1.6 µg of HA equivalent PR8 whole inactivated influenza virus vaccines. One group of mice were given 1.6 µg of both HA equivalent Aichi and PR8 inactivated influenza vaccines (the DIV group). One group of naïve mice was used as a control. For MNP skin application, mice were shaved and treated with hair remover lotion 2 days prior to the immunization. MNPs were firmly held on the shaved dorsal area for 1 min and then left on the skin for 30 min. At week 4, mice were challenged with 2 × LD_50_ of a reassortant A/Vietnam/1203/2004 H5N1 or 2 × LD_50_ of a reassortant A/Shanghai/2013 H7N9 [a recombinant virus containing HA and NA from A/Shanghai (H7N9) or A/Vietnam (H5N1) with PR8 backbone, designated rSH and rVet, respectively] and their body weights were monitored daily for 14 days (25).

### Passive Transfers of Immune Sera *In Vivo*

Mice were primed and boosted with 5 µg HA equivalent of PR8 and Aichi inactivated vaccines at week 0 and week 3, respectively. One group of mice was immunized with 4M2e-tFliC MNPs after the boosting immunization. Three weeks after 4M2e-tFliC MNPs immunization (week 9 after the primary vaccination), two groups of inactivated vaccine immunized mice were intraperitoneally (IP) injected with 200 µl of naïve serum or 4M2e-tFliC immune serum 2 h before 2 × LD_50_ of rVet and rSH virus challenges. One group of naïve mice were injected with 4M2e-tFliC serum as a control. 4M2e-tFliC immune serum was collected and pooled at 3 weeks after mice receiving the 4M2e-tFliC MNPs immunization.

### Cell Lines, Viruses, and Vaccines

Madin–Darby canine kidney (ATCC) cells were cultured as described previously (26). Mouse-adapted influenza A/PR/8/34 (H1N1, PR8), A/Aichi/2/68 (H3N2, Aichi), rSH, and rVet were grown and titrated in the lab (27). The 4M2e-tFliC fusion protein was designed with four different M2e sequences from four different viruses: A/California/07/2009 (H1N1, CA09), A/Aichi/2/68 (H3N2, Aichi), A/Avian/Washington/2014 (H5N1), and A/Avian/Shanghai/2013 (H7N9). Production and determination of the whole inactivated influenza virus vaccines (PR8 and Aichi) and the 4M2e-tFliC fusion protein was done as previously described (23).

### Fabrication of MNPs to Administer Tri-Component Influenza Vaccine

Microneedle patches containing 100 solid, conical microneedles (250-µm diameter at the base and 650-µm long) were fabricated using a two-step molding process on polydimethylsiloxane (PDMS) molds. The first filling solution was a mixture containing 0.5 μg/μl A/Aichi, 0.5 μg/μl A/PR8, 1 µg/µl 4M2e-tFliC, 1% (w/w) sodium carboxymethyl cellulose (CMC-Na), and 10% (w/w) sucrose in 100 mM dibasic potassium phosphate buffer pH 7.4, which was prepared by mixing the different components in the desired ratios. This solution was cast on PDMS molds under vacuum to facilitate filling the MN cavities with the solution. After 30 min, excess solution was removed. The filled molds were then left under vacuum for another 20 min. The second filling solution, containing 18% (w/w) PVA and 18% (w/w) sucrose, was then cast on the filled PDMS molds. This solution was dried under vacuum for another 3 h and then further dried at 35°C overnight before demolding the patches. The patches were immediately stored with desiccant in individually sealed pouches before application. The stability and delivery efficiency of MNPs *in vivo* were determined as previously described (23).

### Determination of Humoral Immune Responses

Blood samples were collected at 3 weeks post-immunization. The levels of Aichi, PR8, and M2e-specific IgG and IgG isotype (IgG1 and IgG2a) antibody titers were measured by ELISA using 4 µg/ml of purified Aichi and PR8 viruses or synthetic M2e peptides as coating antigens, respectively. Mice bronchoalveolar lavage fluids (BALF) and lungs were collected 5 days post challenge, and the measurement of M2e-specific IgG titers in mouse BALF was done by ELISA. Hemagglutinin inhibition (HAI) titers were performed following the WHO manual (28).

### Characterization of Cellular Immune Responses and Lung Viral Titers

Mice were sacrificed at week 4 post-immunization. Spleens and bone marrow were collected and processed into single-cell suspensions with complete RPMI media as previously described (29). Antigen-specific IL-4-, IL-2-, and IFN-γ-secreting cells and virus-specific antibody-secreting cells (ASCs) were determined by an ELISPOT assay as previously described (23).

Inguinal lymph nodes (ILNs) and spleens were collected 7 days post-immunization for the FACS evaluation of germinal center (GC) reactions, including CD4^+^ follicular T helper cells and B cells. 10^6^/100 μl diluted single-cell suspensions were stained with CD4-PE-Cy7, CXCR5-FITC, B220-APC, and GL-7-FITC antibodies for 30 min on ice. Single-cell suspensions from ILNs were also used for the detection of migratory dendritic cells (DCs) with MHCII-PE, CD11c-APC, Langerin-PE, CD11b-Percp/Cy5.5, and CD103-FITC antibodies. The isolation of mouse skin lymphocytes was conducted following a previously described protocol (30). Antibodies were purchased from BD Biosciences and BioLegend. Cells were extensively washed and analyzed by a Fortessa Flow cytometer (BD Biosciences).

Lungs were collected 5 days post challenge. The lung homogenates were prepared for viral titer detection *via* plaque assay as described previously and calculated with the Reed–Muench method (26).

### Detection of Cytokine Secretion *In Vitro*

Single-cell suspensions of ILNs were cultured *in vitro* with PBS and 4 µg/ml of the 4M2e-tFliC fusion protein or PR8 inactivated virus as stimulators for 5 days at 37°C. The supernatants were collected 5 days later to determine the IL-2, IL-4, IL-6, IL-12/p40, and IL-17A levels by cytokine ELISA. Briefly, 96-well plates (MaxiSorp, Nunc) were coated with LEAF™ Purified anti-mouse IL-2, IL-4, IL-6, IL-12/p40, or IL-17A antibodies (BioLegend) at 4°C overnight. After blocking, 100 µl supernatant was added and incubated at 37°C for 2 h. Plates were then washed with PBST and incubated with Biotin anti-mouse IL-2, IL-4, IL-6, IL-12/p40, or IL-17A antibodies (BioLegend) at 37°C for 1 h. After washing with PBST, plates were incubated with streptavidin-HRP (BioLegend) for 1 h and treated with TMB substrate (Thermo Fisher Scientific) for reaction and subsequently 0.18 M H_2_SO_4_ for termination.

### Statistical Analysis

A Shapiro–Wilk Normality test was employed to check the data distribution. A two-tailed Student’s *t*-test was performed to compare the difference significance between two groups if data showing normal distribution. Otherwise, a Mann–Whitney *U* test was used. Statistical results were shown in each figure. A *p* value <0.05 was considered to be statistically significant. *p* < 0.05 (*), *p* < 0.01 (**), *p* < 0.001 (***), *p* > 0.05 (n.s).

## Results

### Evaluation of Humoral Immune Response

The efficacy of skin vaccination *via* dissolving MNP was compared with traditional intramuscular (IM) route. The dissolving MNP encapsulated 3.2 µg of the 4M2e-tFliC fusion protein and 1.6 µg of each component of the DIV (PR8 and Aichi) (tri-component). One IM vaccine group received the same dose of the tri-component vaccine. Another IM vaccine group received only the DIV.

**Figure 1:**
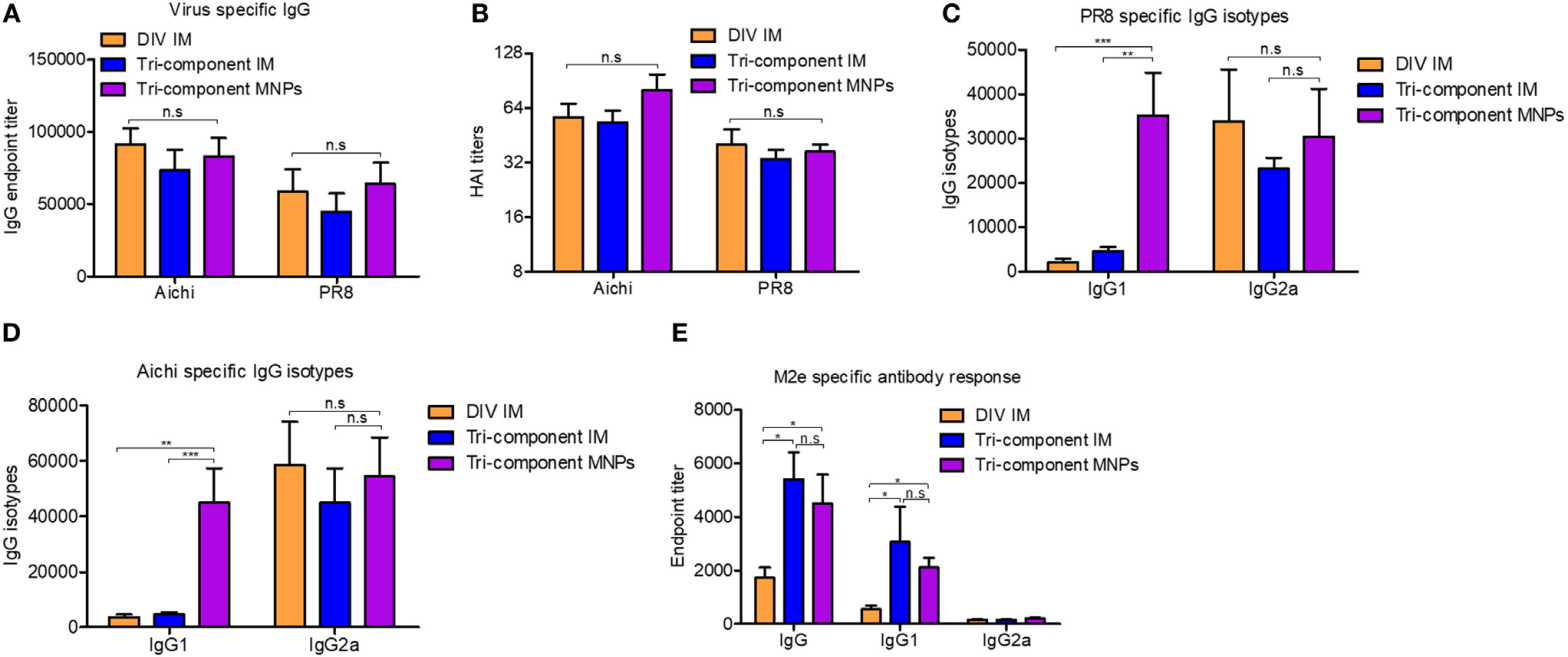
Evaluation of humoral immune response. BALB/c mice were intramuscular (IM) immunized with Aichi and PR8-inactivated bi-component vaccine [divalent inactivated vaccine (DIV)], the combination of the 4M2e-tFliC fusion protein, Aichi and PR8-inactivated tri-component vaccine, or microneedle patch (MNP) skin vaccination with the tri-component vaccine. Serum samples (*N* = 8) were collected 3 weeks post immunization and analyzed for levels of Aichi-specific IgG **(A)** and IgG isotypes **(D)**, PR8-specific IgG **(A)** and IgG isotypes **(C)** and M2e-specific IgG and IgG isotypes **(E)**. **(B)** Hemagglutinin inhibition titers against Aichi and PR8. Data represent mean ± SEM. The statistical significance was analyzed by Mann–Whitney *U* test. *p < 0.05, **p < 0.01, ***p < 0.001, ^n.s^*p* > 0.05.

Mice immunized with MNPs or IM showed similar levels of Aichi- or PR8-specific IgG endpoint titers and HAI titers at week 3 post-immunization (*p* > 0.05) (Figures 1A,B). IgG1 and IgG2a could indicate the Th2 and Th1 type cell responses, and then we also detected the levels of these virus-specific IgG isotypes. We observed significantly pronounced IgG1 titers in MNP-immunized mice (PR8 and Aichi-specific IgG1/IgG2a ratio = 1.158 and 0.812) compared with the tri-component IM group (PR8 and Aichi-specific IgG1/IgG2a ratio = 0.198 and 0.103) or the DIV IM group (PR8 and Aichi-specific IgG1/IgG2a ratio = 0.063 and 0.063) (Figures 1C,D). The DIV and the tri-component IM immunizations induced a Th1-biased immune response. The supplementation of 4M2e-tFliC to the DIV increased the ratio of the IgG1/IgG2a through MNPs immunization. Meanwhile, immunization with the tri-component vaccine with MNPs elicited a more balanced IgG1/IgG2a ratio, which means MNPs immunization induced strong Th1 cell responses and potent Th2 cell responses as well.

We also determined the levels of M2e-specific IgG and IgG isotype-specific antibody titers. Compared with the DIV IM immunized group, the tri-component vaccine delivered *via* MNPs or IM promoted significantly higher M2e-specific IgG and IgG1 titers. Only low amounts of IgG2a were detected in all the groups (Figure 1E). Meanwhile, we observed no differences in M2e-specific IgG or IgG1 levels between the MNPs and IM groups that were immunized with the tri-component vaccine. These results were consistent with our previous data which demonstrated that immunization with 4M2e-tFliC fusion protein alone elicited an IgG1 predominant immune response (23). The observation that 4M2e-tFliC alone MNPs induced higher M2e-specific IgG1, and immunization with the tri-component MNPs induced a much higher level of virus-specific IgG1, indicated that the MNP-based vaccine tended to induce a more balanced IgG1 and IgG2a responses.

### Induction of Cellular Immune Responses

**Figure 2:**
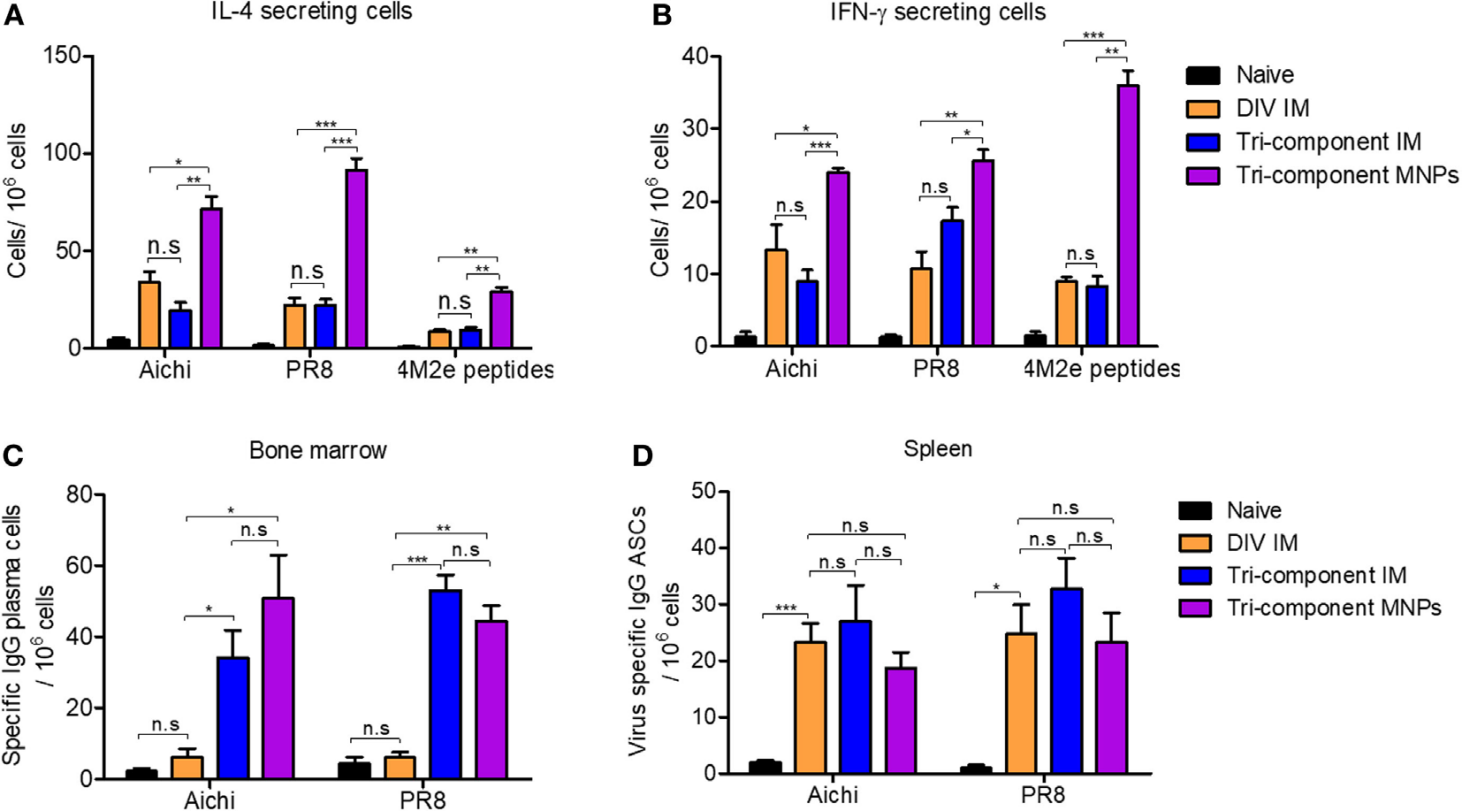
Cellular immune response after the immunization. Splenocytes and bone marrow cells were isolated from mice (*N* = 4) at week 4 post vaccination. **(A)** IL-4-secreting cells in spleens and **(B)** IFN-γ-secreting cells in spleens. Cytokine-secreting cells in splenocytes were measured in the presence of 4 µg/ml purified inactivated Aichi virus, PR8 virus, or M2e peptides as stimulators by the ELISPOT method. **(C,D)** Numbers of Aichi- and PR8-specific IgG plasma cells in bone marrow and spleens, respectively. Data represent mean ± SEM. The statistical significance was analyzed by Student’s *t*-test. *p < 0.05, **p < 0.01, ***p < 0.001, ^n.s^*p* > 0.05.

We measured IL-4 and IFN-γ secreting cells by cytokine ELISPOT 4 weeks after immunizations. After stimulation with Aichi or PR8 virus, we observed significantly elevated numbers of IL-4- and IFN-γ-secreting spleen cells in the MNP-immunized group versus either the DIV or tri-component IM immunized groups. Mice receiving the MNP immunization also showed much more IL-4- and IFN-γ-secreting cells in the spleen after stimulation with M2e peptides versus the IM groups (Figures 2A,B). The IM vaccination groups had similar numbers of IL-4- and IFN-γ-secreting spleen cells. Meanwhile, we detected equal amounts of IL-2-secreting cells within splenocytes from all immunized groups when using inactivated Aichi or PR8 viruses or M2e peptides as stimulators (Figure S1A in Supplementary Material). The increased levels of IL-4-secreting cells in the MNP-immunized group provide evidence that skin vaccination with MNP-encapsulated tri-component vaccine enhances Th2 immune responses.

IL-4 not only plays important roles in Th2 cell differentiation but also facilitates the proliferation and differentiation of B cells into antibody-secreting plasma cells (31–34). Therefore, we evaluated the spleen ASCs after the primary immunization, as well as the bone marrow plasma cells which derived from circulating B plasma cells and behaved independently of IL-4 (35). As shown in Figure 2C, Aichi and PR8 virus-specific IgG plasma cells in bone marrow were elevated 4 weeks after tri-component MNP or IM immunization. However, the number of virus-specific IgG plasma cells in the DIV group was as low as in the naïve group. Similarly, we observed more virus-specific IgA plasma cells in the tissues of mice receiving the tri-component vaccine (Figure S1B in Supplementary Material). Unlike the responses in bone marrow, we observed no significant differences between any of the groups in the numbers of Aichi or PR8-specific IgG ASCs in splenocytes (*p* > 0.05) (Figure 2D). These results show 4M2e-tFliC plays a role in supporting the stimulation of virus-specific bone marrow plasma cells.

### MNP Skin Vaccination Enhanced GC Reactions

**Figure 3:**
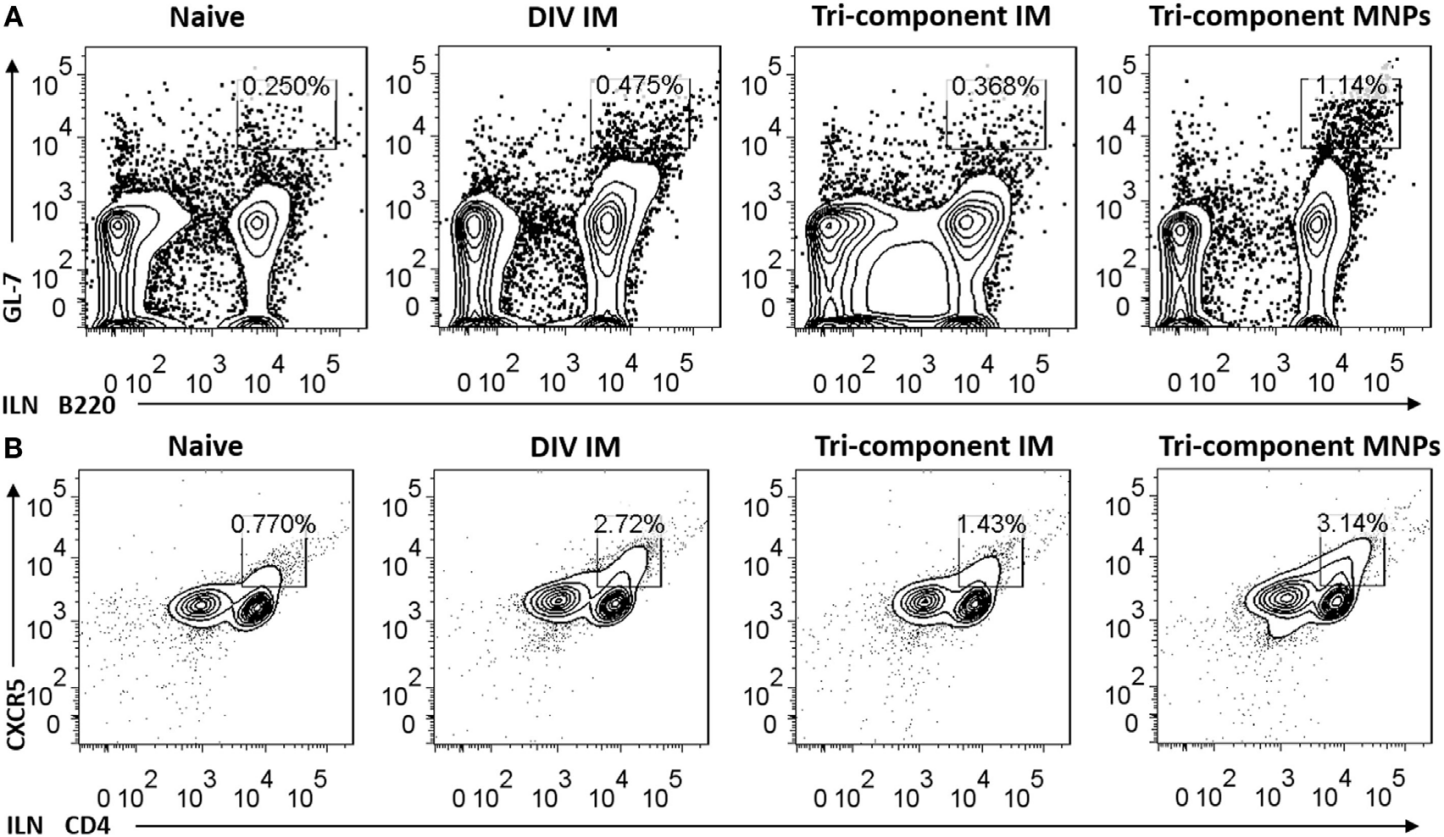
Microneedle patch immunization induced germinal center (GC) reactions in the draining lymph node. The number of GC B cells **(A)** and Tfh cells **(B)** from inguinal lymph nodes was determined 7 days post immunization by FACS (*N* = 4). Data represent mean ± SEM. The statistical significance was analyzed by Student’s *t*-test. *p < 0.05, **p < 0.01, ***p < 0.001, ^n.s^*p* > 0.05.

Germinal center responses stimulate the development of long-lived plasma cells which are critical for the promotion of long-term immune memory. We analyzed the activation of B cells and follicular B helper T cells (Tfh) from ILNs, 7 days post-immunization. We detected a threefold increase in the frequency of B220 and GL-7 double-positive B cells in ILNs from the MNP group when compared with either of the IM groups (Figure 3A). We also detected an elevated frequency of double-positive CD4 and CXCR5 cells in the MNP-immunized mice compared with tri-component IM group (Figure 3B). We also determined the numbers of B220^+^GL-7^+^ B cells and CD4^+^CXCR5^+^ T cells in splenocytes. We observed a higher frequency of B220^+^GL-7^+^ splenocytes in the tri-component IM immunized mice (Figure S2 in Supplementary Material).

To exclude the possibility that the enhanced GC reactions were caused by the MNP device, two groups of mice were immunized with either PBS or 4M2e-tFliC MNPs to test the GC responses in ILNs 7 days post-immunization. As shown in Figures S3A,B in Supplementary Material, compared with the PBS MNP-immunized group, the 4M2e-tFliC MNP induced an approximately 3.5- and 4-fold increase in the numbers of B220^+^GL-7^+^ B cells and CD4^+^CXCR5^+^ T cells, respectively. These results show that MNP-based immunization is a potent strategy to induce GC reactions.

### Induction of Cytokine Responses *In Vitro*

The secretion levels of IL-2, IL-4, IL-6, IL-12/p40, and IL-17A from the ILN lymphocytes are indicators of innate and cellular immune responses. We analyzed these cytokines for a better understanding of the immune responses to MNP immunization. At day 7 after the immunizations, we re-stimulated lymphocytes isolated from ILNs with 4M2e-tFliC fusion proteins or inactivated PR8 for 5 days to determine the cytokines secreted into the supernatant *in vitro*.

**Figure 4:**
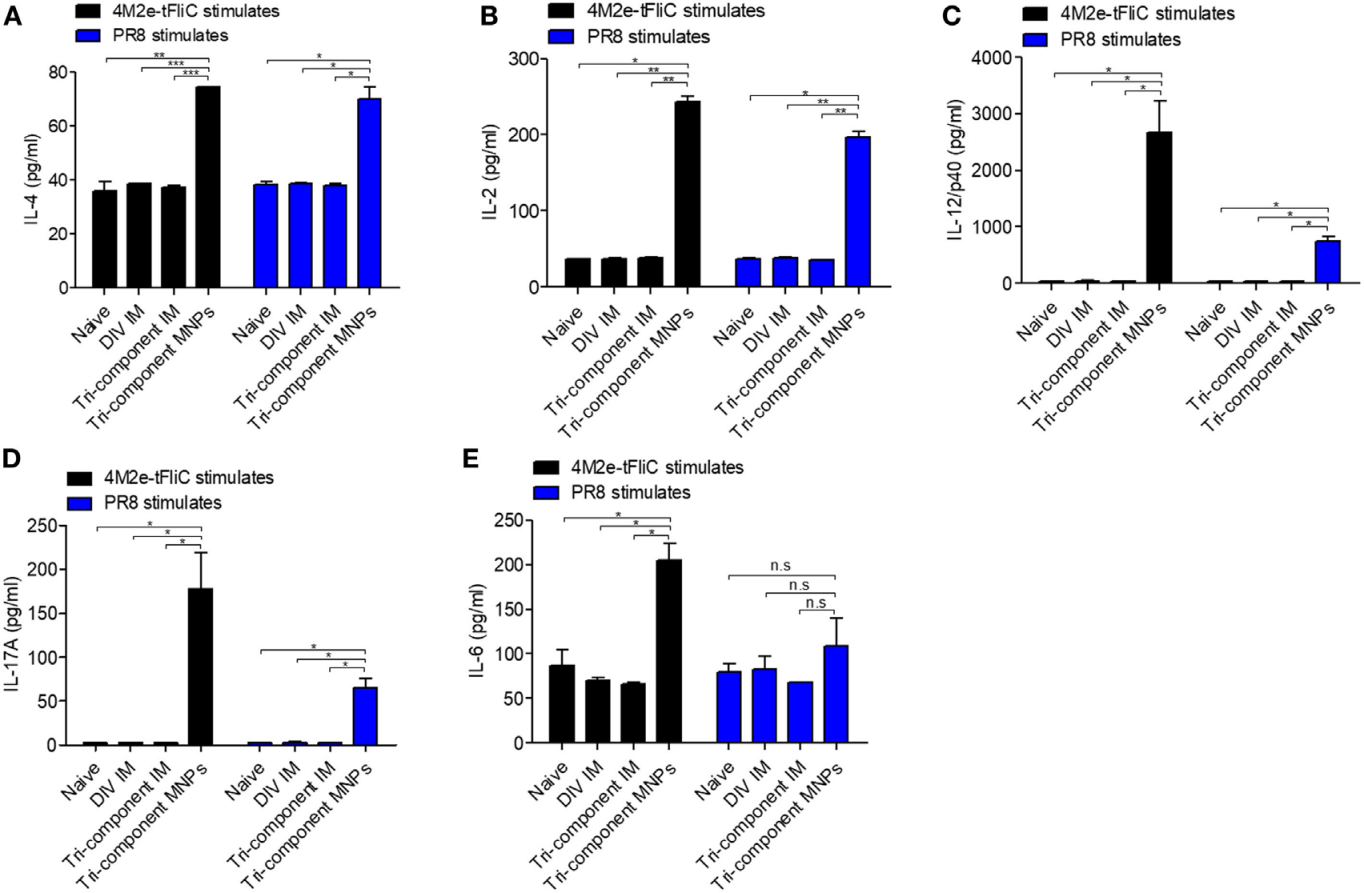
Induction of cytokine secretion *in vitro*. Single-cell suspensions from inguinal lymph nodes (*N* = 3) were stimulated with 4M2e-tFliC fusion protein or PR8-inactivated virus *in vitro* for 5 days. The cytokines in supernatants were determined by cytokine ELISA. **(A)** IL2; **(B)** IL-4; **(C)** IL-12/P40; **(D)** IL-17; **(E)** IL-6. Data represent mean ± SEM. The statistical significance was analyzed by Student’s *t*-test. *p < 0.05, **p < 0.01, ***p < 0.001, ^n.s^*p* > 0.05.

We observed twofold higher levels of IL-4 after 4M2e-tFliC or inactivated PR8 stimulation in the MNP-immunized group versus the IM immunized groups or naïve group (Figure 4A). We also observed higher levels of IL-2 in the MNP-immunized group after stimulation (Figure 4B). The most striking differences in the cytokines released after stimulation we observed were the significant levels of IL-12/p40 and IL-17A levels in the MNP-immunized group. In sharp contrast, the levels of IL-12/p40 and IL-17A cytokines in the IM groups or naïve group were very low (Figures 4C,D).

IL-17A is involved in the mediation of proinflammatory responses by inducing production of many other cytokines, such as IL-6. As shown in Figure 4E, we detected higher levels of IL-6 in the MNP group following 4M2e-tFliC fusion protein stimulation, but we observed no difference following stimulation by inactivated PR8. Meanwhile, we observed increased secretion of IL-12/p40 and IL-17A from cell suspensions of ILNs at day 7, but not day 4, after 4M2e-tFliC MNP immunization (Figure S4 in Supplementary Material).

Overall, these data provide evidence indicating that by creating a mildly inflammatory environment in draining lymph nodes, MNP immunization induces more comprehensive immune responses than the traditional IM injection, such as elevated GC B cell and Tfh cell responses.

### Inhibition of Heterologous Influenza Virus Infection

**Figure 5:**
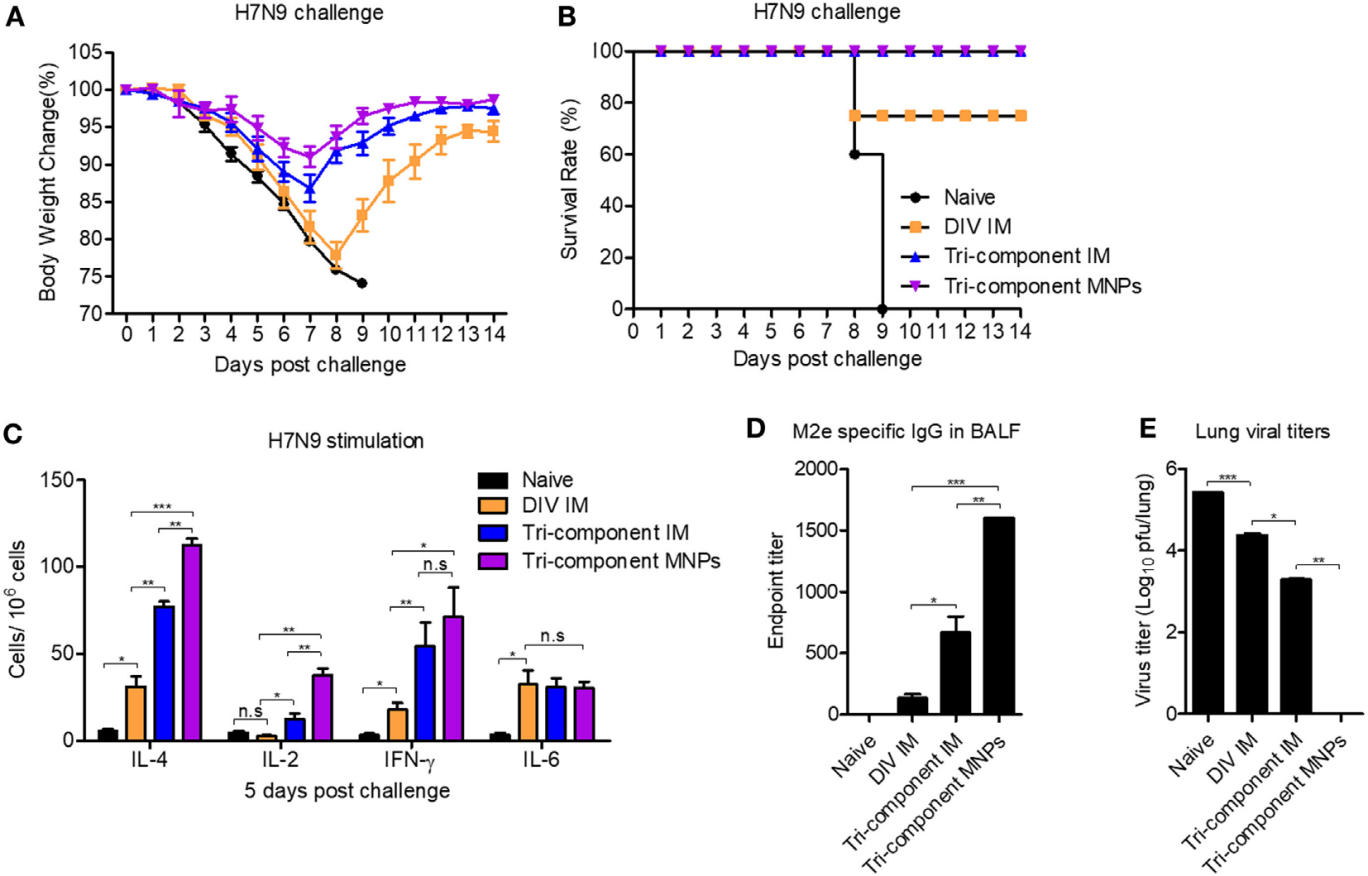
Protection efficacy against a heterologous influenza virus challenge. BALB/c mice (*N* = 5) were intramuscular (IM) immunized with divalent inactivated vaccine (DIV), tri-component vaccine, or microneedle patch (MNP) immunized with the tri-component vaccine. Four weeks after the immunization, mice were challenged with 2 × LD_50_ of A/Shanghai/02/2013 (H7N9, rSH) reassortant virus. Body weight changes **(A)** and survival rates **(B)** were monitored daily for 14 days. Mice were sacrificed at day 5 post challenge for the collection of spleens, bronchoalveolar lavage fluids (BALF), and lungs. **(C)** Cytokine-secreting cells in splenocytes were measured in the presence of 4 µg/ml purified A/Shanghai/02/2013 (H7N9, rSH) virus by ELISPOT. **(D)** The levels of M2e-specific IgG in BALF. **(E)** Lung viral titers. Data represent mean ± SEM. The statistical significance was analyzed by Student’s *t*-test. *p < 0.05, **p < 0.01, ***p < 0.001, ^n.s^*p* > 0.05.

We have demonstrated that the tri-component MNP vaccination augmented immune responses versus the other vaccines tested. To compare the protective efficacy against avian influenza virus infection, we immunized groups of mice and challenged them with 2 × LD_50_ of the reassortant A/Shanghai/2013 (H7N9, rSH) influenza virus. As shown in Figures 5A,B, after rSH infection, all naïve mice reached their endpoints within 9 days. The DIV IM immunization provided 60% protection, and mice were severely sick. Compared with the DIV IM group, the tri-component MNP and IM vaccines provided 100% protection against H7N9 virus challenge with mice showing 10 and 15% body weight loss, respectively (Figures 5A,B).

We sacrificed mice at day 5 post rSH infection and measured the cellular immune responses in the spleen. Compared with the DIV IM group, there were greater numbers of IL-2-, IL-4-, and IFN-γ-secreting cells in the tri-component immunized groups following stimulation with inactivated rSH. We detected the largest numbers of IL-2- and IL-4-secreting cells in the MNP-immunized group (Figure 5C). No significant differences were observed among the immunization groups in the numbers of IL-6-secreting cells (Figure 5C).

In agreement with the observed cellular immune responses in the spleen, the tri-component immunizations enhanced M2e-specific IgG antibody titers in BALF 5 days after rSH infection. We detected over two times higher levels of M2e-specific IgG antibody titers in the BALF of mice from the MNP-immunized group versus the tri-component IM group (Figure 5D). Due to the improved cellular immune responses in the spleen and the humoral immune responses in the BALF, we measured significantly lower levels of lung viral titers from the tri-component immunized mice. We detected the lowest levels of viral titers in the MNP-immunized group (Figure 5E).

These results show that immunization with 4M2e-tFliC fusion protein improved immune responses and increased inhibition of virus infection. MNPs fabricated with 4M2e-tFliC and inactivated influenza vaccines amplified the already enhanced immune responses and provided extra protection against the heterologous avian influenza viral infection.

### 4M2e-tFliC MNP Immune Sera Confer Protection in Heterologous Virus Infection

We performed passive administration of 4M2e-tFliC MNP immune serum to determine the protective role of M2e-specific antibodies. We collected 4M2e-tFliC MNP immune sera from vaccinated mice and pooled them together at 3 weeks after the immunization. The detailed immunization method was described in Section “Materials and Methods” and a diagram of the experimental strategy was included in Supplementary Material (Figure S6 in Supplementary Material).

We primed and boosted three groups of mice with inactivated PR8 and Aichi at week 0 and week 3, respectively. Three weeks after the boosting immunization, we immunized one group of mice with 4M2e-tFliC MNPs. At 3 weeks after the 4M2e-tFliC MNPs immunization (week 9 after the primary vaccination) and 2 h before A/Vietnam/1203/2004 (H5N1, rVet) or rSH reassortant virus challenges, we IP injected the other two groups of inactivated influenza immunized mice with naïve serum or 4M2e-tFliC immune serum. We injected one group of naïve mice with 4M2e-tFliC serum as a control.

**Figure 6:**
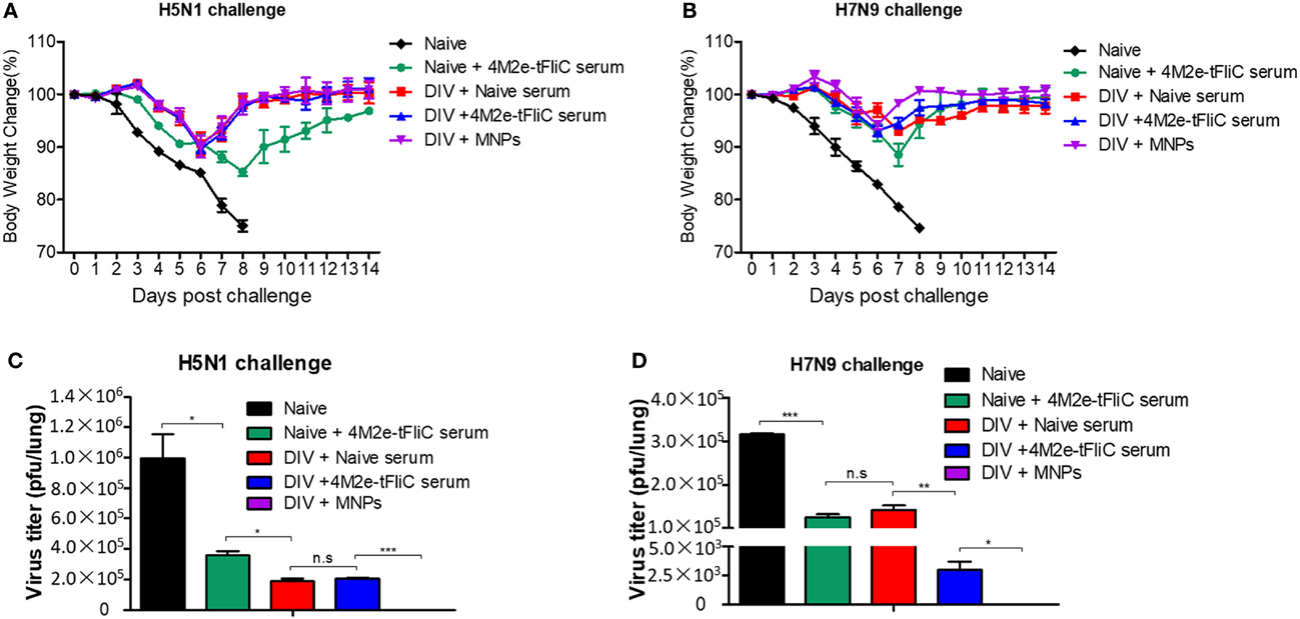
4M2e-tFliC microneedle patch (MNP) immune sera play a protective role in heterologous virus infection. BALB/c mice (*N* = 5) were intramuscular (IM) immunized twice with the divalent inactivated vaccine (DIV). One group of immunized mice was boosted with 4M2e-tFliC MNPs for the third immunization (DIV + MNPs). Three weeks after the MNP vaccination, the other two groups of DIV-immunized mice were intraperitoneally (IP) injected with naïve serum (DIV + Naïve serum) or 4M2e-tFliC immune serum (DIV + 4M2e-tFliC serum), respectively. Naïve mice received 4M2e-tFliC immune serum as a control (Naïve + 4M2e-tFliC serum). **(A,B)** Body weight change after 2 × LD_50_ of A/Vietnam/1203/2004 (H5N1, rVet) and A/Shanghai/02/2013 (H7N9, rSH) reassortant virus challenges. **(C,D)** Lung viral titers at day 5 post H5N1 and H7N9 challenges. Data represent mean ± SEM. The statistical significance was analyzed by Student’s *t*-test. *p < 0.05, **p < 0.01, ***p < 0.001, ^n.s^*p* > 0.05.

As shown in Figures 6A,B, 4M2e-tFliC immune serum passive transfer decreased lung viral titers and provided 100% protection to naïve mice that were infected with 2 × LD_50_ of H5N1 or H7N9 influenza viruses. We also observed that mice receiving the 4M2e-tFliC immune serum showed significantly decreased H7N9 viral titers in lungs compared with similar mice, naïve, or immunized, which received the naïve serum (Figure 6D).

We detected much lower levels of virus in 4M2e-tFliC MNP-immunized mice versus all other groups (Figures 6C,D). This demonstrates that besides the inhibition function of the M2e-specific antibodies, the cellular immune responses induced by the MNP and the heterologous prime-boost were critical for the protective efficacy of this vaccination strategy.

### Mechanisms Underlying MNP Skin Immunization

**Figure 7:**
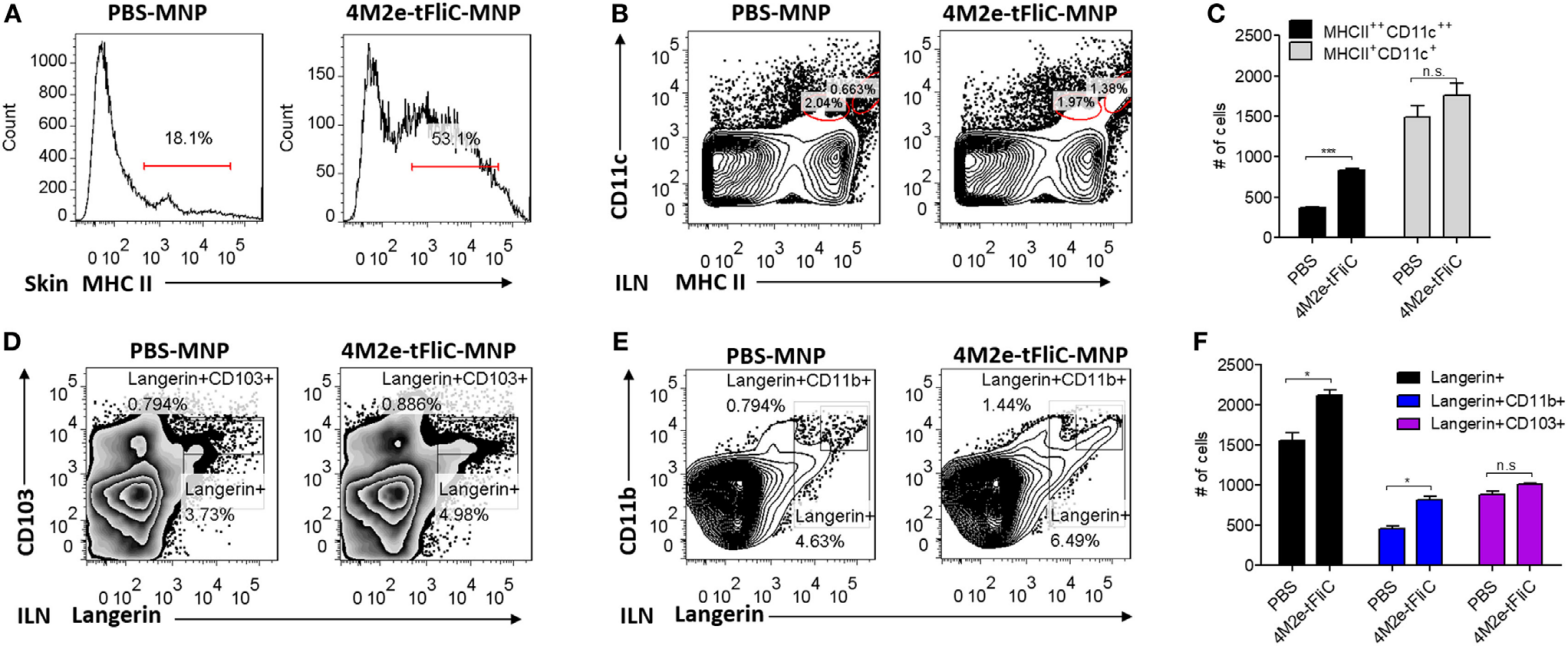
Microneedle patch (MNP) skin vaccination promoted antigen-presenting cell (APC) recruitment and dendritic cells (DCs) migration. **(A)** MHC II-positive cells were detected from mouse skin lymphocytes at day 7 post PBS treatment or 4M2e-tFliC MNP skin vaccination. The frequencies **(B)** and the numbers **(C)** of migratory DCs and subsets of migratory DCs **(D–F)** in inguinal lymph nodes were determined at day 7 post vaccination by FACS (*N* = 4). Data represent mean ± SEM. The statistical significance was analyzed by Student’s *t*-test. *p < 0.05, **p < 0.01, ***p < 0.001, ^n.s^*p* > 0.05.

To further understand the immunological value of MNP vaccination, we immunized mice with PBS or 4M2e-tFliC MNP. We aimed to determine the early phase cellular immune responses after 4M2e-tFliC MNP immunization. Seven days post-immunization, we collected skin tissue and ILNs to analyze the frequency of different cell populations. As shown in Figure 7A, 53.1% MHCII^+^ cells could be detected at the skin immunization site of the mice 7 days post-immunization compared with the 18.1% MHC II^+^ cells observed in the PBS MNP-immunized group.

Dendritic cells are the primary antigen-presenting cells (APCs) that initiate T cell responses. We observed a significantly increased population of migratory MHC II^++^ CD11c^++^ DCs in the skin-draining lymph nodes (ILN) after 4M2e-tFliC MNP immunization (Figures 7B,C). Langerin is an important marker of epidermal and dermal DCs. We could detect increased numbers of Langerin^+^ migratory DCs and a greater percentage of Langerin^+^CD11b^+^ cells in the skin-draining lymph nodes of the 4M2e-tFliC MNP-immunized mice (Figures 7D–F). Meanwhile, compared with soluble 4M2e-tFliC IM or PBS MNP immunization, 4M2e-tFliC MNP vaccination elicited an increased percentage of GC B cells and CD4^+^ Tfh cells in ILNs (Figures S5 and S3 in Supplementary Material).

MHC II is highly upregulated in the process of the APCs maturation. Therefore, these results show that 4M2e-tFliC MNP immunization recruited MHC II^+^ APCs to the immunization site and stimulated the migration of Langerin^+^ DCs to skin-draining lymph nodes and induced strong GC reactions.

## Discussion

This study shows that skin vaccination with a tri-component influenza vaccine using an MNP can generate robust humoral and cellular immune responses and provide better protection than the traditional IM injection. Besides the immunological advantages, the MNP vaccination platform possesses many logistical advantages over traditional vaccines, such as ease of use (e.g., mass vaccination and self-administration), avoidance of biohazardous sharps waste, and independence from the cold chain (17–20). These characteristics make MNPs an excellent candidate for a potential universal influenza vaccine.

Our work and other previous reports have shown that MNPs containing a monovalent influenza vaccine provided 100% protection against the homologous virus and limited protection against heterologous virus infection (29, 36, 37). A recent clinical trial showed MNP vaccination generated robust immune responses, had an excellent safety profile, and had strong acceptability by study participants (38). Also, MNPs containing M2e-based vaccines have been shown to provide better cross-protection against heterologous virus infections (26, 39). In this study, we combined an M2e-based vaccine with two subtypes of inactivated influenza vaccines and fabricated them into a single MNP with the aim of developing a feasible universal influenza vaccine. Compared with the traditional IM injection, the tri-component vaccine delivered by MNP induced increased immunological responses and better protective efficacy against heterologous avian influenza virus infection. The results demonstrate the advantages of MNP-based skin vaccination. In this study, PR8 and Aichi inactivated viruses represent different subtypes of influenza A virus, and these two subtypes are the major subtypes that spread during the flu seasons. PR8 and Aichi can be replaced by any predicted seasonal influenza strains. We hope this newly designed vaccine could be used as a model for the design of future universal influenza vaccine.

The skin has been shown to be an attractive target for vaccine administration due to its high concentration of APCs. These APCs can be divided into several populations according to the expression of Langerin and their location in the skin structure. These divisions include epidermal Langerhans cell (LCs, Langerin^+^CD11b^+^), Langerin^+^ dermal DCs (Langerin^+^XCR1^+^CD11b^low^, Langerin^+^XCR1^+^CD103^+/−^), Langerin^−^ dermal DCs (Langerin^−^CD11b^+/low^), dermal macrophages, and monocyte-derived DC (21, 40, 41). Migration to the LNs and interaction with naïve T cells are basic features of the epidermal LCs and dermal DCs. MNPs contain 100 or more tiny microneedles that penetrate the epidermis and reach the dermis with only minor disturbance of the vasculature. This shallow dermal penetration allows for MNP-based vaccines to fully exploit the potential of dermal APCs for better antigen processing and presentation. Our observations—of significantly increased numbers of cells highly expressing MHC II in the inoculated skin and highly double-positive MHC II/CD11c cells in the skin-draining LNs—support the application of MNPs.

We detected a population of Langerin^+^CD11b^+^ migratory DCs and a higher number of activated CD4^+^ follicular helper T cell and GC B cells in the skin-draining LNs at day 7 post 4M2e-tFliC MNP skin vaccination. These data suggest that the increased humoral and cellular immune responses might be due to the maturation of Langerin^+^ CD11b^+^ skin DCs (LCs), the migration of LCs to the ILN, and LCs interaction with and activation of the CD4^+^ T cells in the draining lymph nodes.

However, more detailed information regarding which subsets of migratory DCs was induced by the MNP vaccine is needed. Uncovering the regulatory mechanisms of MNP vaccination on skin DC maturation and migration is important for targeting specialized subsets of skin DCs to elicit different T cell responses. For example, targeting antigens to XCR1^+^ conventional dendritic cells with anti-Clec9A antibodies in mice enabled strong CD8 and CD4 T cell responses (42, 43). In another example, intradermal immunization with lentivectors activated skin dermal dendritic cells to induce CD8^+^ T cell responses (44). Also, the protective effector memory T (T_EM_) cells generated by skin vaccination can enter the skin to induce local T cell responses for protection against skin infections (45). These observations support that well-designed MNP-based vaccines induce robust and broad T cell responses for protection against a range of different infections.

Skin DCs are a bridge between the innate and adaptive immunity. Activated CD4^+^ T cells can be stimulated by different cytokines secreted by skin DCs to differentiate into diverse subtypes, including Th1, Th2, Th17, and Treg. APC-derived IL-12 plays key roles in the development of Th1 cell responses with the production of IL-2 and IFN-γ, while Th2 cell differentiation heavily depends on T-cell-derived IL-4 (46, 47). The balanced high levels of humoral IgG1 and IgG2a antibody titers in serum of the MNP-immunized group correlated with high ratios of IL-4- versus IL-2- and IFN-γ-secreting cells in the draining LNs and the spleen. Meanwhile, the increased production of IL-12/p40 and IL-4 *in vitro* after stimulation with the 4M2e-tFliC fusion protein or inactivated PR8 suggest the formation of both Th1 and Th2 subtypes.

Previous research has shown that IL-1β, IL-6, IL-23, and TGF-β might be critical components for supporting Th17 differentiation (48). Th17 cells are characterized by the production of cytokines (IL-17A, IL-6, and IL-22), the ability to recruit neutrophils and macrophages, and the ability to regulate inflammation reactions (47, 49). The increased *in vitro* secretion levels of IL-6, IL12/p40, and IL-17A in lymphocytes from skin-draining LNs show that our tri-component MNP vaccine upregulated Th17 differentiation. In addition, LCs have been shown to be essential for the induction of antigen-specific Th17 responses (50, 51). Therefore, Th17 cell responses may participate in immune responses after 4M2e-tFliC MNP skin vaccination. Further studies are required to deduce the regulatory mechanisms involved.

Several approaches could be explored to improve the interactions between antigens encapsulated in MNPs and skin DCs to maximize the desired immune responses. For example, nanoparticles composed of protein or DNA could be an efficient way to optimize DC targeting by MNP vaccines (52–54). Nanoparticle-based vaccines with components directly targeting markers on DC surfaces, like the TLR and NOD ligands, can further enhance the immunogenicity of the antigens within the nanoparticles (55, 56). TLRs are expressed on many cells in the skin, including TLR5, which is known to recognize bacterial FliC and activate innate immunity (57). Thus, the FliC in the 4M2e-tFliC fusion protein delivered *via* MNP may target this specific subset of dermal DCs and contribute to the increased immune responses we observed.

The frequent human infection of the avian H7N9 strain could escalate into a pandemic when it experiences future genetic drift or shift. This possibility highlights the urgency to develop a more broadly effective and rapidly distributable influenza vaccine. The vaccination strategy in this study takes advantage of MNP skin vaccination, M2e immunity, the immunogenicity of inactivated influenza vaccines and FliC’s adjuvant effect. We demonstrated that mice receiving the combination of the 4M2e-tFliC fusion protein and the two types of inactivated influenza *via* MNP skin vaccination are an effective approach to generate extra protection against heterologous avian influenza virus infection.

## Ethics Statement

All animal experiments were performed in accordance with the protocol (Protocol number A16029) approved by Georgia State University’s Institutional Animal Care and Use Committee (IACUC). This study was in strict compliance with the Animal Welfare Act Regulations, the Public Health Service (PHS) Policy on Humane Care and Use of Laboratory Animals, and the Guide for the Care and Use of Laboratory Animals.

## Author Contributions

WZ, MP, and B-ZW designed research. WZ, SL, and CW performed research. WZ, SL, GY, and B-ZW analyzed the data. WZ, MP, and B-ZW wrote the paper.

## Conflict of Interest Statement

MP is an inventor of patents licensed to companies developing microneedle-based products, is a paid advisor to companies developing microneedle-based products, and is a founder/shareholder of companies developing microneedle-based products (Micron Biomedical). This potential conflict of interest has been disclosed and is managed by Georgia Tech and Emory University. All other authors have no potential conflict of interest.
