# Supplementary material for: Enhanced Immune Responses Conferring Cross-Protection by Skin Vaccination With a Tri-Component Influenza Vaccine Using a Microneedle Patch
Source: Front Immunol. 2018 Jul 30;9:1705. doi: 10.3389/fimmu.2018.01705 (PMC6077188; doi:10.3389/fimmu.2018.01705)
Supplement: Supplementary file 1 [file presentation_1.pptx]

## Slide 1
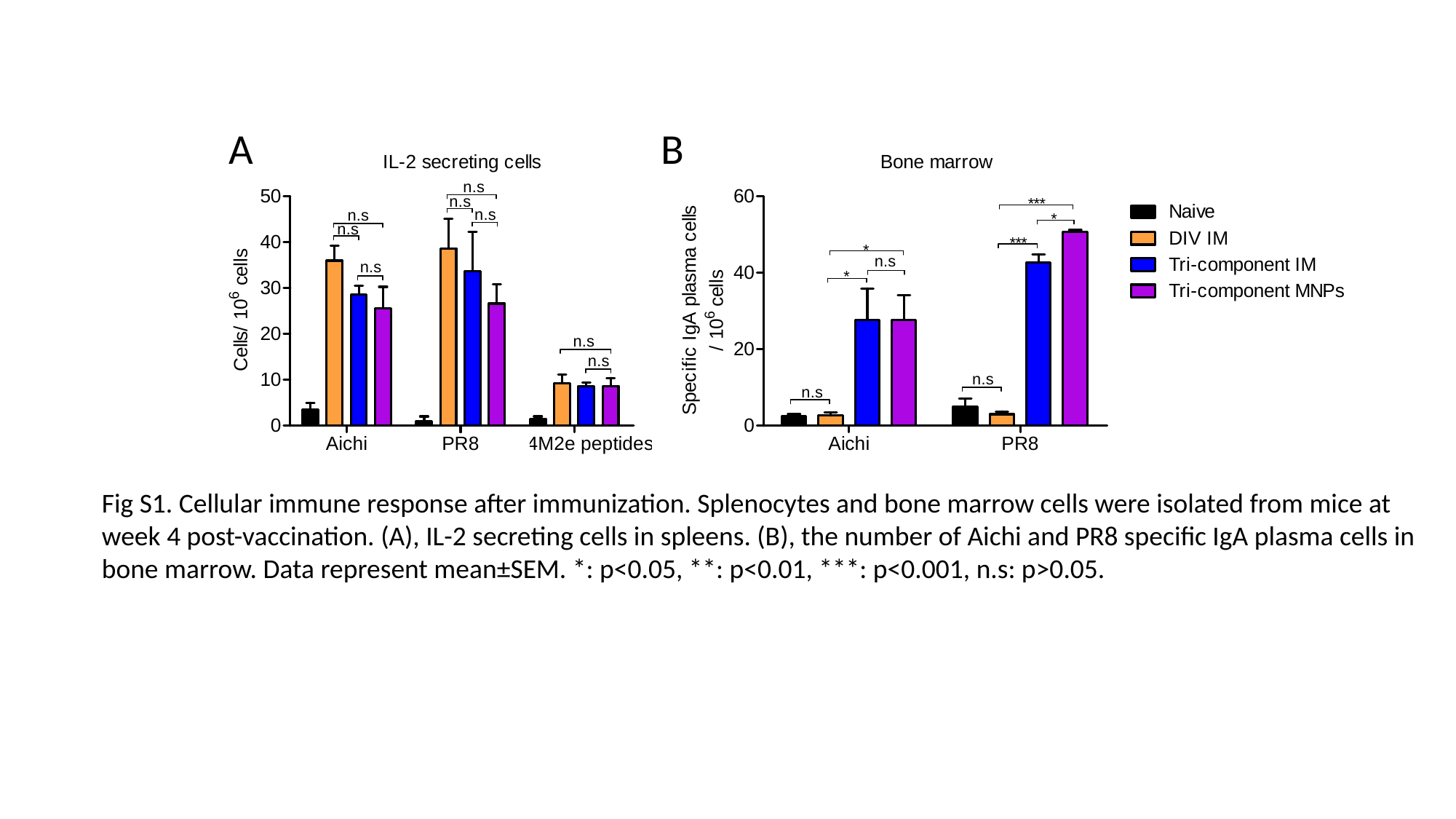

A
B
Fig S1. Cellular immune response after immunization. Splenocytes and bone marrow cells were isolated from mice at week 4 post-vaccination. (A), IL-2 secreting cells in spleens. (B), the number of Aichi and PR8 specific IgA plasma cells in bone marrow. Data represent mean±SEM. *: p<0.05, **: p<0.01, ***: p<0.001, n.s: p>0.05.

## Slide 2
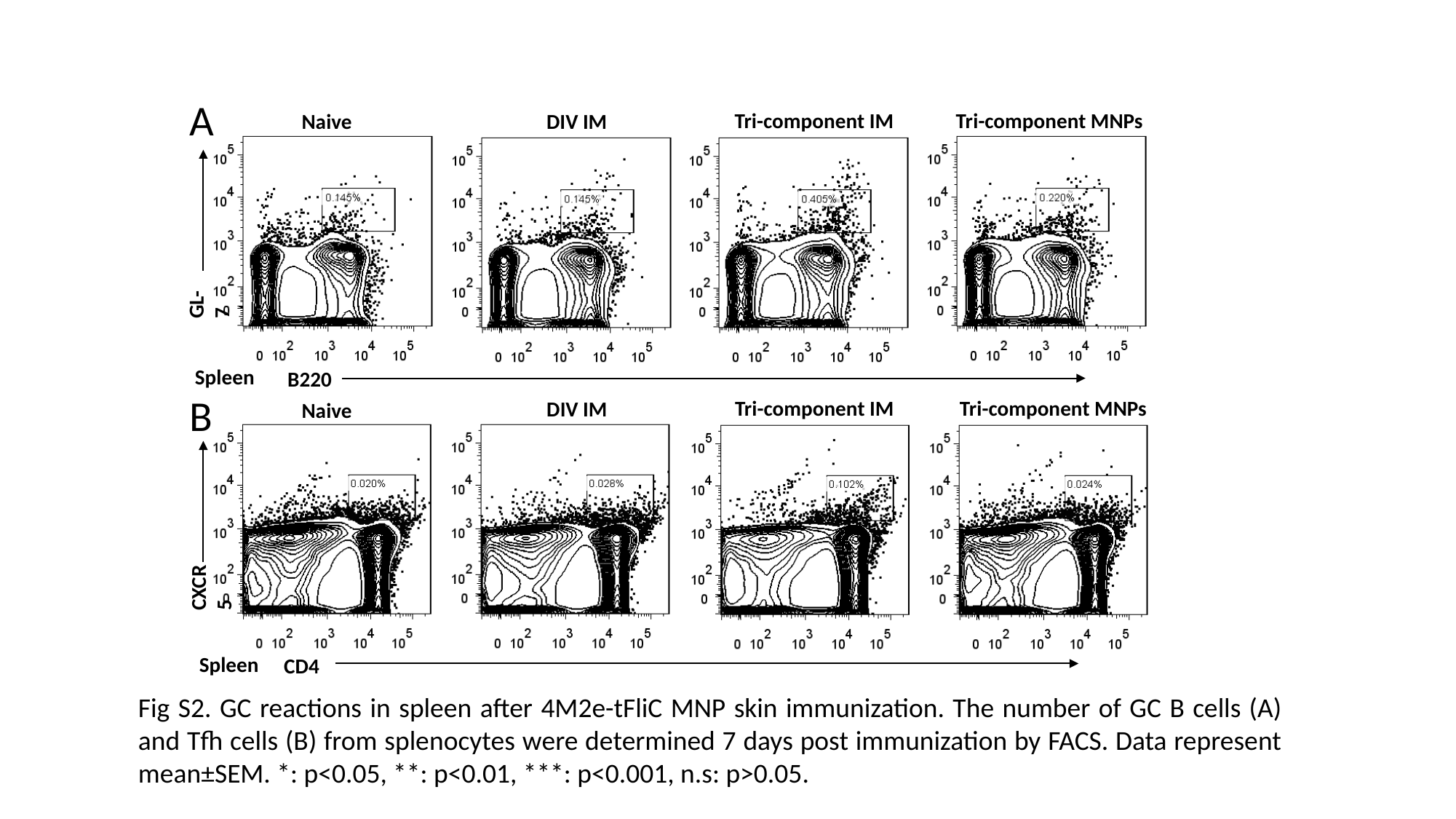

A
Tri-component MNPs
Tri-component IM
DIV IM
Naive
GL-7
Spleen
B220
B
Tri-component MNPs
Tri-component IM
DIV IM
Naive
CXCR5
Spleen
CD4
Fig S2. GC reactions in spleen after 4M2e-tFliC MNP skin immunization. The number of GC B cells (A) and Tfh cells (B) from splenocytes were determined 7 days post immunization by FACS. Data represent mean±SEM. *: p<0.05, **: p<0.01, ***: p<0.001, n.s: p>0.05.

## Slide 3
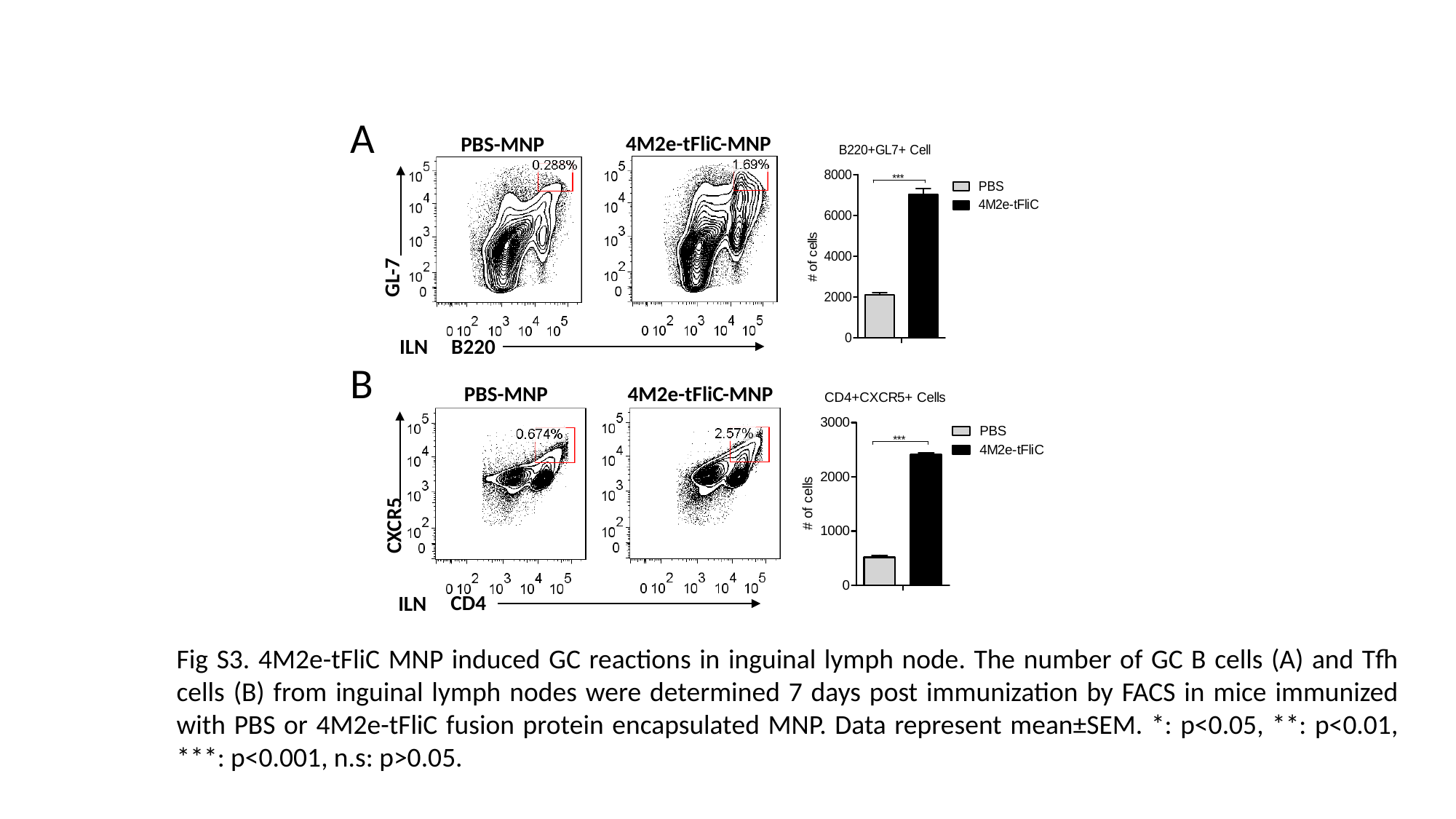

A
4M2e-tFliC-MNP
PBS-MNP
GL-7
ILN
B220
B
PBS-MNP
4M2e-tFliC-MNP
CXCR5
CD4
ILN
Fig S3. 4M2e-tFliC MNP induced GC reactions in inguinal lymph node. The number of GC B cells (A) and Tfh cells (B) from inguinal lymph nodes were determined 7 days post immunization by FACS in mice immunized with PBS or 4M2e-tFliC fusion protein encapsulated MNP. Data represent mean±SEM. *: p<0.05, **: p<0.01, ***: p<0.001, n.s: p>0.05.

## Slide 4
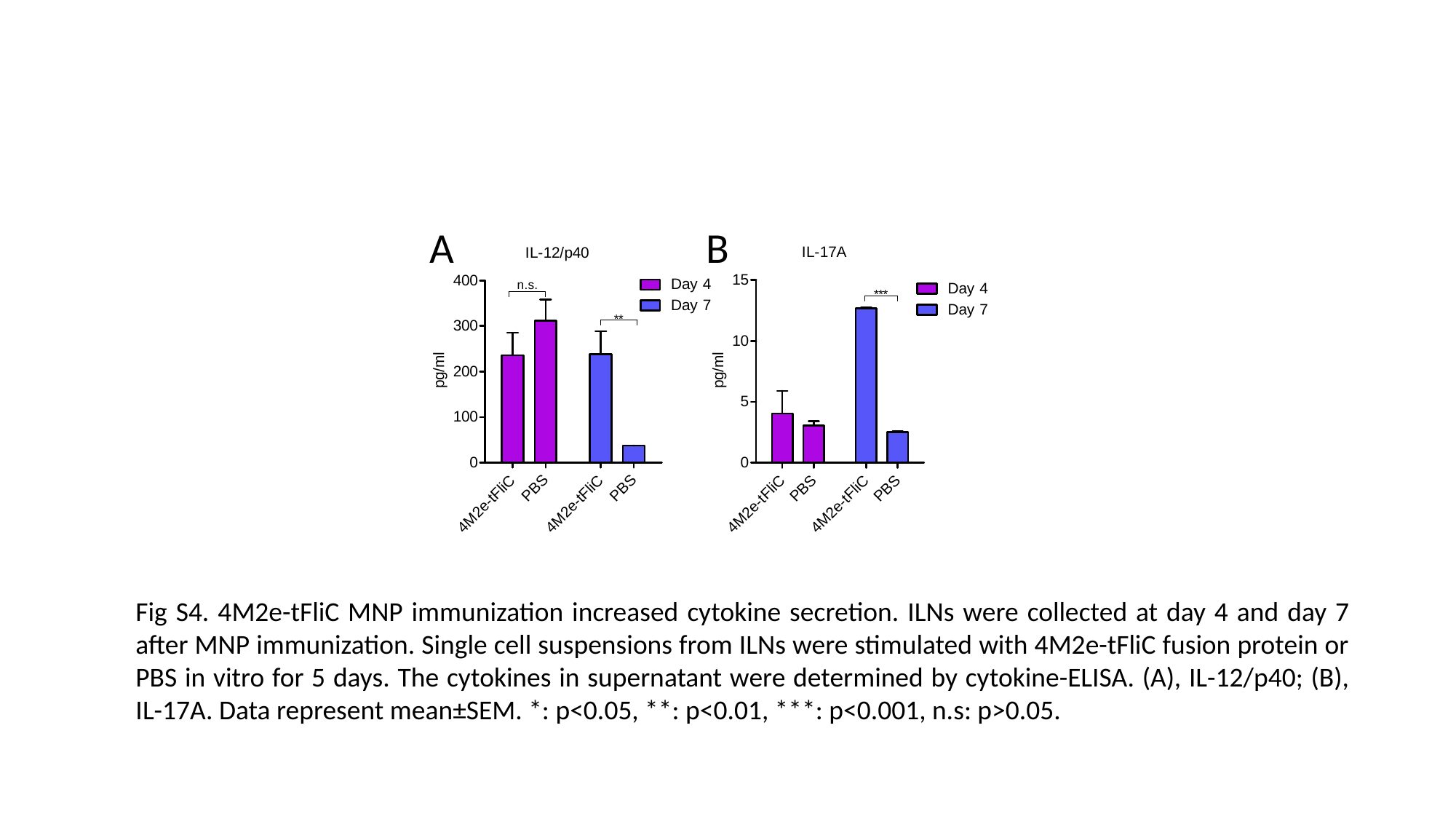

A
B
Fig S4. 4M2e-tFliC MNP immunization increased cytokine secretion. ILNs were collected at day 4 and day 7 after MNP immunization. Single cell suspensions from ILNs were stimulated with 4M2e-tFliC fusion protein or PBS in vitro for 5 days. The cytokines in supernatant were determined by cytokine-ELISA. (A), IL-12/p40; (B), IL-17A. Data represent mean±SEM. *: p<0.05, **: p<0.01, ***: p<0.001, n.s: p>0.05.

## Slide 5
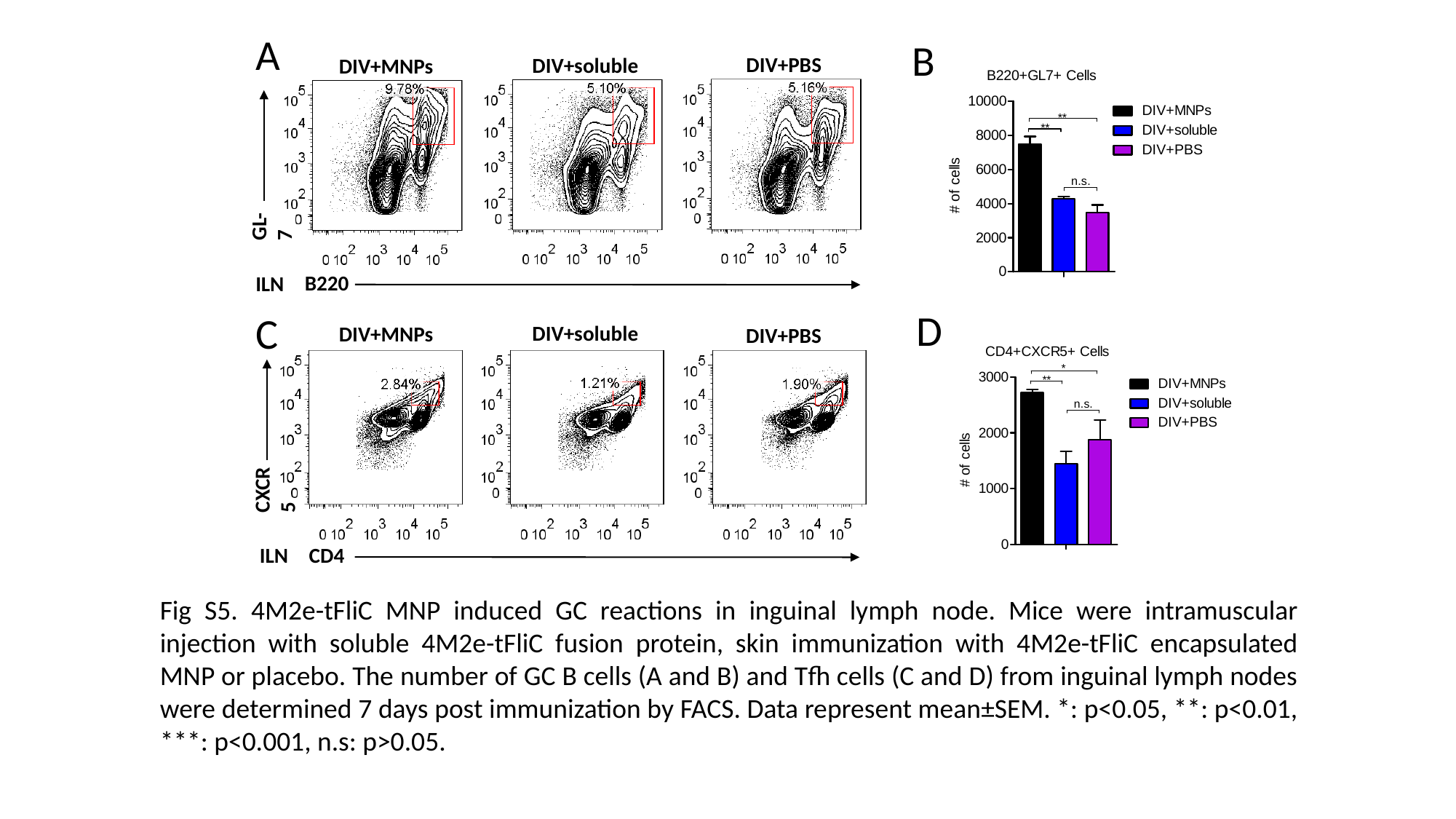

A
DIV+PBS
DIV+soluble
DIV+MNPs
GL-7
B220
ILN
B
D
C
DIV+soluble
DIV+MNPs
DIV+PBS
CXCR5
CD4
ILN
Fig S5. 4M2e-tFliC MNP induced GC reactions in inguinal lymph node. Mice were intramuscular injection with soluble 4M2e-tFliC fusion protein, skin immunization with 4M2e-tFliC encapsulated MNP or placebo. The number of GC B cells (A and B) and Tfh cells (C and D) from inguinal lymph nodes were determined 7 days post immunization by FACS. Data represent mean±SEM. *: p<0.05, **: p<0.01, ***: p<0.001, n.s: p>0.05.

## Slide 6
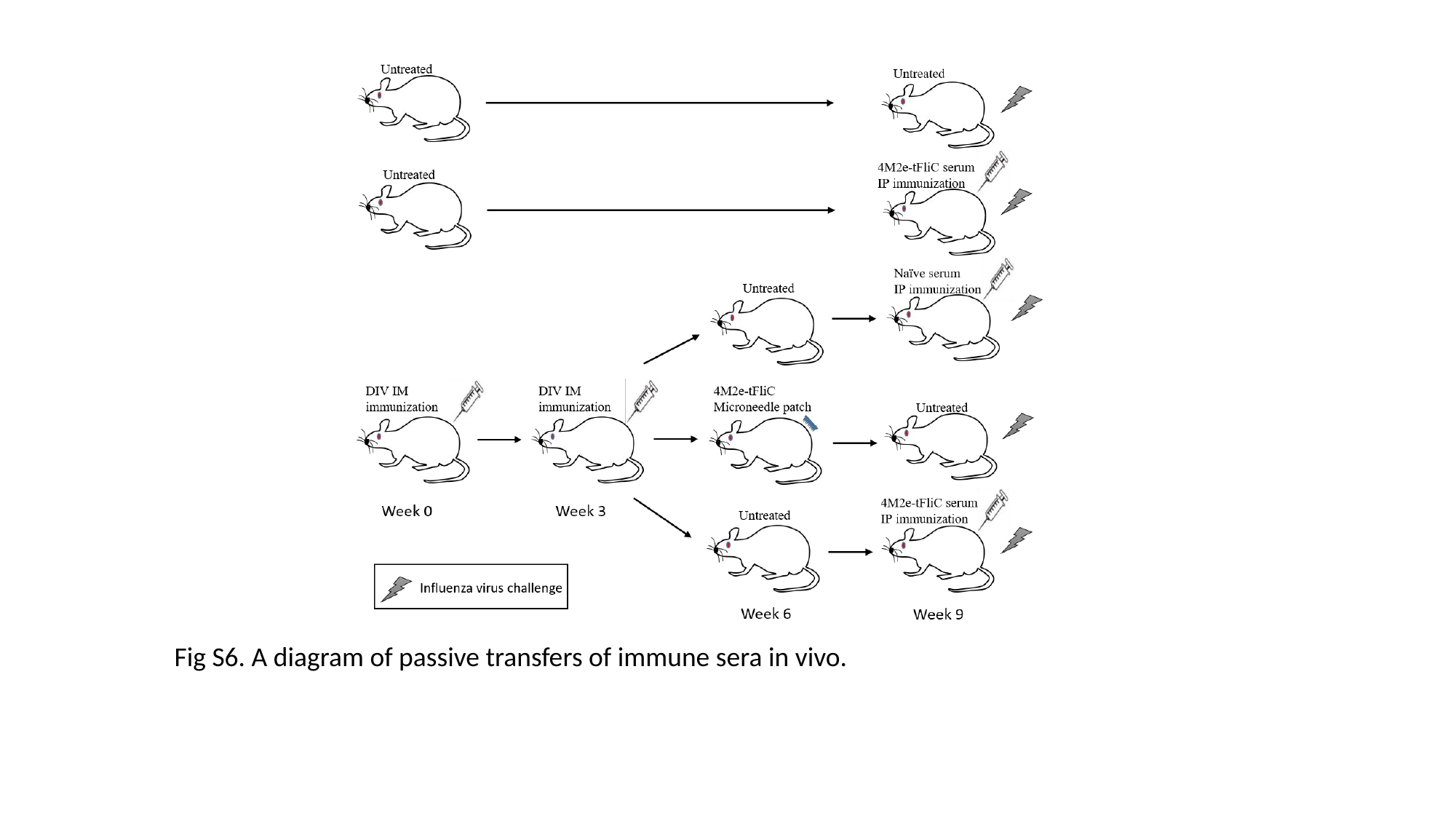

Fig S6. A diagram of passive transfers of immune sera in vivo.
